# Perimedullary arteriovenous fistula was misdiagnosed as intervertebral disc herniation: A case report

**DOI:** 10.1097/MD.0000000000031079

**Published:** 2022-10-14

**Authors:** Zhengbo Yuan, Fengzhen Xiong, Zefu Li

**Affiliations:** a Department of Neurosurgery, Binzhou Medical University Hospital, No. 661 Huanghe 2nd Road, Binzhou, Shandong, 256603, China.

**Keywords:** spinal perimedullary arteriovenous fistula, misdiagnosis, intervertebral disc herniation

## Abstract

Spinal perimedullary arteriovenous fistula (PMAVFS) is a rare intradural vascular malformation with a high rate of misdiagnosis. In adults, most spinal PMAVFs are small and low-flow, starting with progressive spinal dysfunction.

**Patient concerns:**

The patient was a 58-year-old male who presented with both lower limbs numb with intermittent walking weakness, obvious at both ankles, and no obvious inducing and relieving factors. The local hospital considered the diagnosis of lumbar disc herniation after MR examination; he was treated with lumbar fixation and fusion.

**Diagnosis:**

After admission, a ce-MRA examination showed that the left spinal artery at the T10 level showed small branch blood vessels in the local area. The distal end was unclear, which seemed to be connected with the drainage vein of the spinal cord. The digital subtraction angiography (DSA) result indicated that the left intercostal artery of T10 sent the Adamkiewicz artery down to the level of L4, and an arteriovenous fistula was seen. The fistula was located at the lower edge of the L4 level and then drained to the upper premedullary vein to the level of T4 after a short descending. It was finally diagnosed as a perimedullary arteriovenous fistula.

**Interventions:**

It was cured by cutting the arteriovenous fistula in the spinal canal by indocyanine green-assisted angiography.

**Outcomes:**

we report a case of PMAVFS misdiagnosed as lumbar disc herniation with resection and internal fixation. In our hospital, the final diagnosis was a perimedullary arteriovenous fistula, which was cured by cutting off the arteriovenous fistula within the spinal canthus.

**Conclusion:**

Spinal perimedullary arteriovenous fistula (PMAVFS) is a rare intradural vascular malformation with a high rate of misdiagnosis. In adults, most spinal PMAVFs are small and low-flow, starting with progressive spinal dysfunction. It is hoped that this can provide warnings to more neurosurgeons and reduce the occurrence of misdiagnosis.

## 1. Introduction

Spinal cord perimedullary arteriovenous fistula (PMAVFS) is an intradural vascular malformation, which refers to the pathological connection between the spinal artery and medullary vein without an intermediate nodule, located on the surface of the spinal cord or under the pia mater. It is caused by natural movement without an intermediate lesion.^[1]^ PMAVFS is relatively rare in clinical practice, accounting for 8% to 19% of spinal vascular malformations, and rates of misdiagnosis and missed diagnosis are incredibly high.^[2]^ In adults, most of the spinal cord PMAVF are small and low-flow, starting with progressive spinal cord dysfunction.^[3]^ The treatment methods are mainly surgical resection, endovascular treatment, and a combination of the two methods. Generally, the most suitable treatment methods are selected according to the location of PMAVFS and the patient’s condition. At present, we report that a case of PMAVFS was misdiagnosed as a lumbar disc herniation with resection and internal fixation. In our hospital, it was finally diagnosed as a perimedullary arteriovenous fistula. It was cured by cutting the arteriovenous fistula in the spinal canal by indocyanine green-assisted angiography. The report is as follows:

## 2. Case presentation

**Figure 1.**
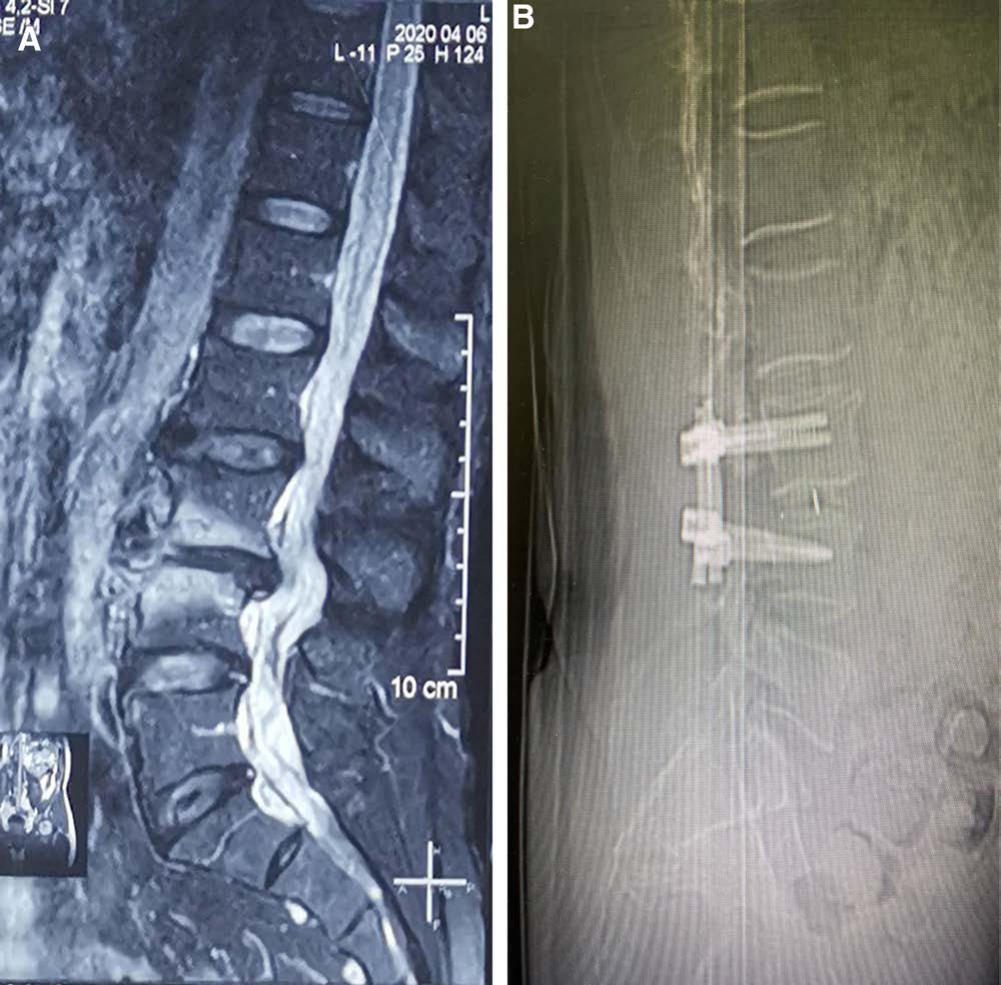
MR (A) before discectomy and fusion in the local hospital, and CT (B) after the operation in the local hospital.

The patient was a 58-year-old male who presented with both lower limbs numb with intermittent walking weakness, obvious at both ankles, and no obvious inducing and relieving factors. The local hospital considered the diagnosis of lumbar disc herniation after MR examination; he was treated with lumbar fixation and fusion; at that time, he was not found with perimedullary arteriovenous fistula (Fig. 1), as there was a little symptomatic improvement after the surgery. After admission to our department, the muscle strength of both lower limbs was grade 5, the dorsal flexor muscle strength was grade 4, and the muscle tension was normal.

**Figure 2.**
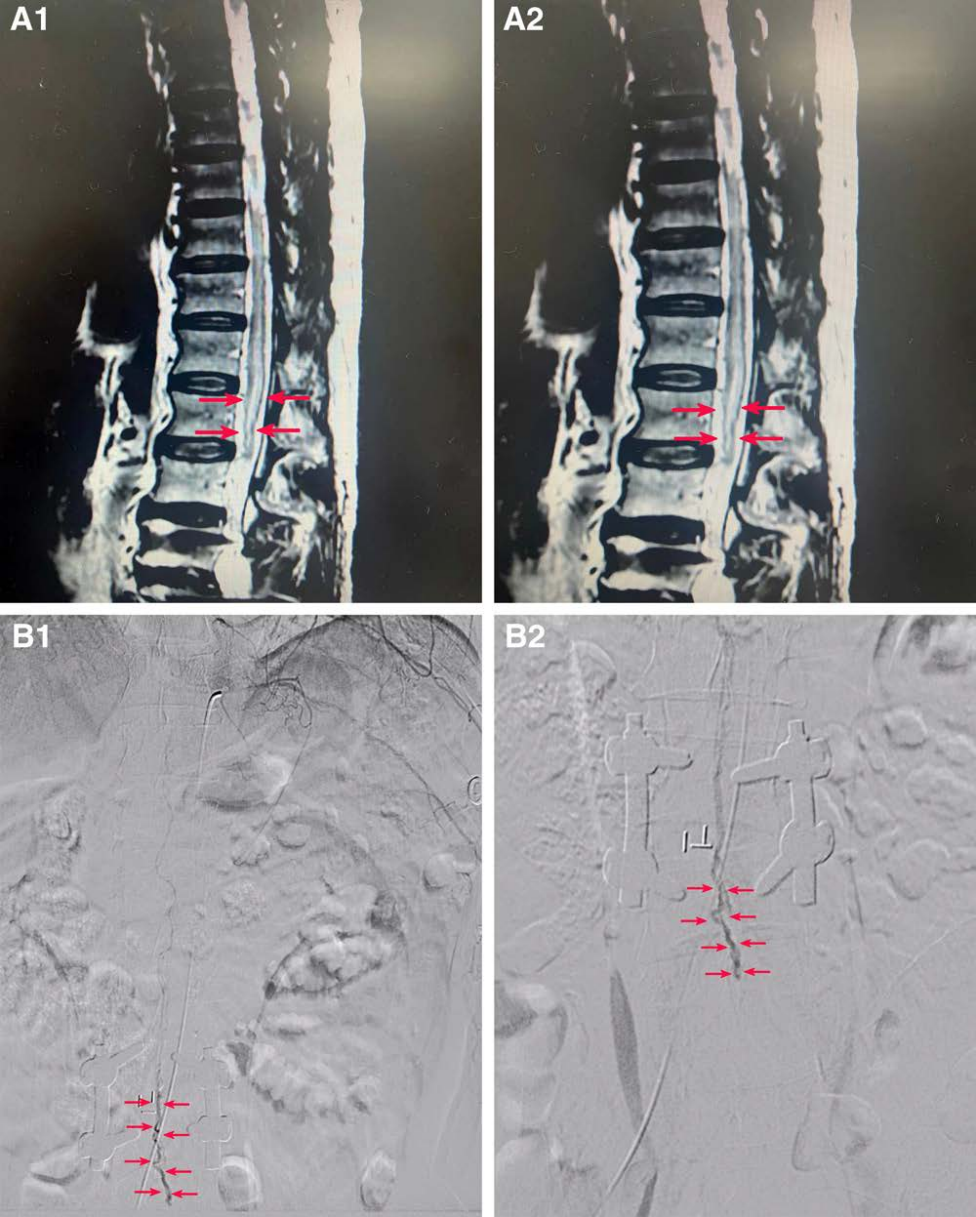
ce-MRA showed that the left spinal dural artery at the T10 level showed small branch blood vessels in the local area, and the distal display was unclear, which seemed to be connected with the drainage vein of the spinal cord (A1–A2). The DSA result indicated that the left intercostal artery of T10 sent the Adamkiewicz artery down to the level of L4, and an arteriovenous fistula was seen. The fistula was located at the level of the lower edge of L4. After a short descending, it drained to the upper premedullary vein to the level of T4 (B1–B2). DSA = digital subtraction angiography.

After admission, a ce-MRA examination showed that the left spinal artery at the T10 level showed small branch blood vessels in the local area. The distal end was unclear, which seemed to be connected with the drainage vein of the spinal cord (Fig. 2A1–A2). The digital subtraction angiography (DSA) result indicated that the left intercostal artery of T10 sent the Adamkiewicz artery down to the level of L4, and an arteriovenous fistula was seen. The fistula was located at the lower edge of the L4 level and then drained to the upper premedullary vein to the level of T4 after a short descending (Fig. 2B1–B2). According to the results of ce-MR and DSA, he was diagnosed with a spinal cord arteriovenous fistula, and we planned the fistula resection under indocyanine green-assisted angiography.

**Figure 3.**
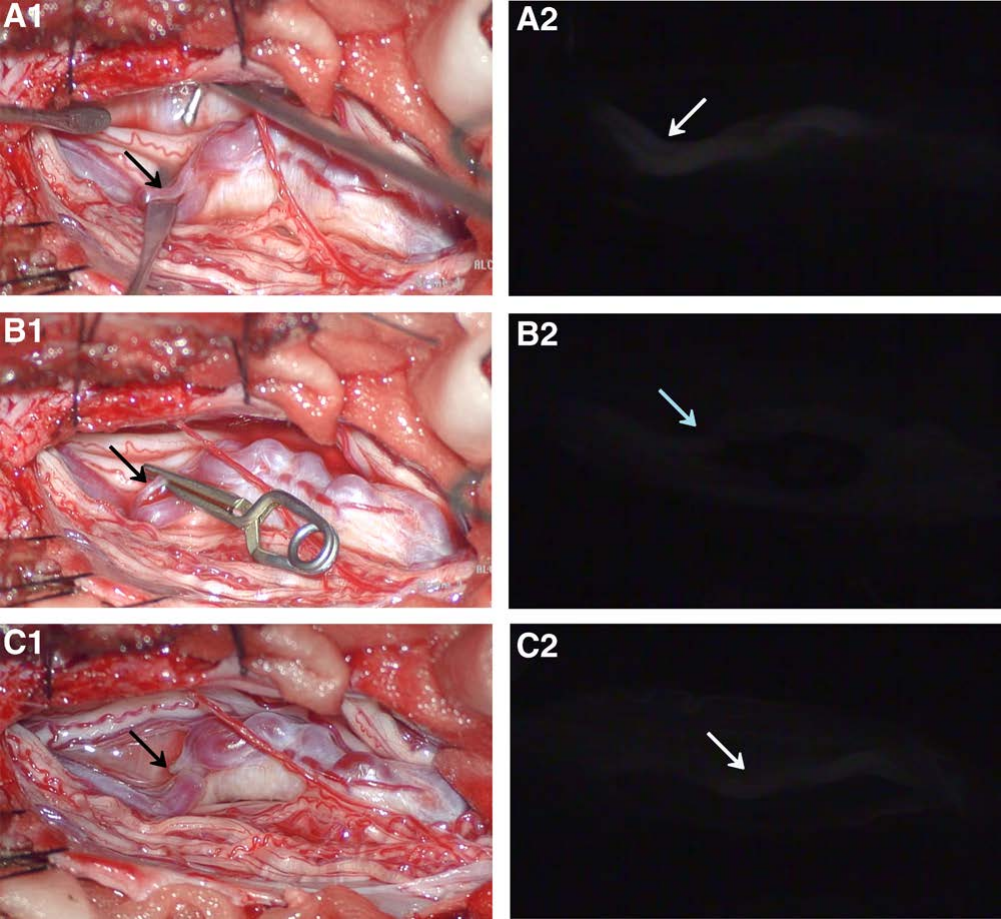
The intraoperative image is combined with the integrated microscope’s indocyanine green (ICG) video angiography image. Microscopic and ICG video angiography images show the location of the dural arteriovenous fistula (A1–A2). After clipping the perimedullary arteriovenous fistula, the visual field under the microscope, and the ICG video angiography image (B1–B2). The field of vision under the microscope and ICG video angiography image after resectioning the perimedullary arteriovenous fistula (C1–C2).

According to the patient’s condition, location, and the size of the fistula, we chose the method of removing the fistula under a microscope. In order to locate the fistula accurately, we performed indocyanine green-assisted imaging in the combined operation room. The approach is first to expose the L4 spinous process and bilateral lamina, bite the L4 spinous process and lamina with the rongeur, expose the normal dura mater, and sharply separate the L3 dorsal scar tissue along the standard interface of the dura mater. The dura mater underneath is exposed, and the dural tension is not high. The dura mater was cut longitudinally to separate the cauda equina nerve. The ventral terminal filament of the cauda equina nerve showed a loop-shaped malformed vascular mass with tortuous vessels. According to the blood supply artery shown by DSA, a slightly thinner and thicker artery was seen upwards. The veins accompany, forming an arteriovenous fistula at the end, approximately swelling at the bottom, and the veins below are tortuous into a mass. Fluorescence contrast was performed with 5 mL indocyanine green to confirm that the artery was supplying the arteriovenous fistula. A temporary blocking clip was used to clamp the blood supply artery. Fluorescence contrast was performed again with 5 mL indocyanine green to confirm the malformed vascular mass. After the development delay fades, the blood supply artery is electrocautery and cut off. Then, indocyanine green angiography showed that the blood supply arteries disappeared, and the venous mass development was delayed. After confirming that the blood supply artery is cut off, the dura mater is tightly sutured after thorough hemostasis and flushing (Fig. 3). The patient was given symptomatic treatment after the operation, and his symptoms improved ten days later.

## 3. Discussion

According to the patient’s condition, location, and the size of the fistula, we chose the method of removing the fistula under a microscope. In order to locate the fistula accurately, we performed indocyanine green-assisted imaging in the combined operation room. The approach is first to expose the L4 spinous process and bilateral lamina, bite the L4 spinous process and lamina with the rongeur, expose the normal dura mater, and sharply separate the L3 dorsal scar tissue along the standard interface of the dura mater. The dura mater underneath is exposed, and the dural tension is not high. The dura mater was cut longitudinally to separate the cauda equina nerve. The ventral terminal filament of the cauda equina nerve showed a loop-shaped malformed vascular mass with tortuous vessels. According to the blood supply artery shown by DSA, a slightly thinner and thicker artery was seen upwards. The veins that accompany PMAVF are entirely different from spinal vascular malformation. According to the recognized Anson and Spetzler classification, it is divided into three subtypes according to the shunt flow and the degree of vasodilation.^[4]^ In type IVa PMAVF, there is a slow-flow single shunt between the undilated anterior or posterolateral spinal artery and spinal vein. Type IVB lesions have more blood flow than type IVa lesions, and the ampullae of the shunt venous side is dilated. The increased shunt can cause dilation and tortuous intradural veins.^[5]^ PMAVFS usually has more than one blood supply artery, the dilated anterior spinal artery, and one or two posterior spinal arteries.^[6]^ The IVC type PMAVFS is known as giant periampullary AVFS. It has multiple high-flow dilated blood supply arteries and large drainage veins.^[7]^

However, the early spinal cord PMAVFS is usually tricky; it can only be diagnosed when the patient has apparent symptoms.^[8]^ At present, the main diagnostic methods are magnetic resonance angiography (MRA),^[9]^ computed tomography angiography (CTA), and DSA. However, digital subtraction angiography is still the gold standard of diagnosis.^[10]^

The goal of the treatment of perimedullary arteriovenous fistula is to completely occlude arteriovenous communication while retaining regular arterial blood supply and venous drainage. At present, the treatment of spinal cord PMAVFS includes endovascular embolization, surgical resection, and a combination of the two methods.^[2]^ In general, the treatment strategy is determined by the patient’s condition, location, and fistula size. Due to the accessibility of the intravascular approach, small terminal filament lesions seem to be a good indication for surgical treatment.^[11]^ However, surgical treatment is also challenging because sometimes it is difficult to find fistulas embedded between congestive veins, and the location and eradication of shunts can become very difficult. Therefore, the key to surgical treatment of spinal cord PMAVFS is locating the fistula and removing or closing the fistula. In this regard, intraoperative near-infrared indocyanine green (ICG) video angiography is an effective method.^[12]^ ICG shows the transition from the spinal artery to the medullary vein, which helps remove or close the fistula. Therefore, ICG was used in this operation to determine the location of the fistula. The use of ICG has been described in a shorter series. The authors concluded that ICG angiography is a simple and effective technique for intraoperative identification of PMAVFS sites and occlusion evaluation.^[13]^ However, the disadvantage of ICG is that the display of the surgical field is indirect, and the magnification is suppressed. Fluorescein seems to be a valuable option in PMAVFS surgery.^[14]^

The clinical recovery of perimedullary arteriovenous fistulas may depend on many factors, such as the initial degree and duration of neurological defects, previous spinal cord injuries caused by bleeding, compressive myelopathy, or chronic congestive myelopathy.^[15]^ On the other hand, reducing vascular theft, the size of the tremendous venous sac and pulse pressure, and the reduction of venous hypertension after treatment may significantly improve the clinic. In this sense, early detection and treatment may be beneficial.^[16]^

## 4. Conclusion

PMAVFS mainly occurs after progressive spinal cord dysfunction, so the rate is exceptionally high for misdiagnosis rate and missed diagnosis. Therefore, it is essential to choose the correct diagnosis method. Indocyanine green-assisted angiography is a suitable method to locate the position of the fistula during the operation for PMAVFS that can be treated by surgical resection.

## Author contributions

ZL performed the surgery in this case. FX and ZY contributed to the treatment of the patient and the writing of this manuscript. All authors have read and approved the final version of the manuscript.

**Conceptualization:** Zefu Li.

**Methodology:** Fengzhen Xiong.

**Resources:** Zhengbo Yuan.

**Supervision:** Zefu Li.

**Writing – original draft:** Zhengbo Yuan.

**Writing – review & editing:** Zhengbo Yuan.
